# Methods for detecting probable COVID-19 cases from large-scale survey data also reveal probable sex differences in symptom profiles

**DOI:** 10.3389/fdata.2022.1043704

**Published:** 2022-11-10

**Authors:** Amit Klein, Karena Puldon, Stephan Dilchert, Wendy Hartogensis, Anoushka Chowdhary, Claudine Anglo, Leena S. Pandya, Frederick M. Hecht, Ashley E. Mason, Benjamin L. Smarr

**Affiliations:** ^1^Department of Bioengineering, University of California, San Diego, La Jolla, CA, United States; ^2^Osher Center for Integrative Health, University of California, San Francisco, San Francisco, CA, United States; ^3^Department of Management, Zicklin School of Business, Baruch College, The City University of New York, New York, NY, United States; ^4^Halicioglu Data Science Institute, University of California, San Diego, La Jolla, CA, United States

**Keywords:** public health, mHealth, sex as a biological variable, Bayesian network, infectious disease

## Abstract

**Background:**

Daily symptom reporting collected via web-based symptom survey tools holds the potential to improve disease monitoring. Such screening tools might be able to not only discriminate between states of acute illness and non-illness, but also make use of additional demographic information so as to identify how illnesses may differ across groups, such as biological sex. These capabilities may play an important role in the context of future disease outbreaks.

**Objective:**

Use data collected via a daily web-based symptom survey tool to develop a Bayesian model that could differentiate between COVID-19 and other illnesses and refine this model to identify illness profiles that differ by biological sex.

**Methods:**

We used daily symptom profiles to plot symptom progressions for COVID-19, influenza (flu), and the common cold. We then built a Bayesian network to discriminate between these three illnesses based on daily symptom reports. We further separated out the COVID-19 cohort into self-reported female and male subgroups to observe any differences in symptoms relating to sex. We identified key symptoms that contributed to a COVID-19 prediction in both males and females using a logistic regression model.

**Results:**

Although the Bayesian model performed only moderately well in identifying a COVID-19 diagnosis (71.6% true positive rate), the model showed promise in being able to differentiate between COVID-19, flu, and the common cold, as well as periods of acute illness vs. non-illness. Additionally, COVID-19 symptoms differed between the biological sexes; specifically, fever was a more important symptom in identifying subsequent COVID-19 infection among males than among females.

**Conclusion:**

Web-based symptom survey tools hold promise as tools to identify illness and may help with coordinated disease outbreak responses. Incorporating demographic factors such as biological sex into predictive models may elucidate important differences in symptom profiles that hold implications for disease detection.

## Introduction

The COVID-19 pandemic has highlighted the need for low-burden public health screening tools. Traditional approaches focus on the use of aggregated hospital records for observational studies of disease progression (Pham et al., 2017; Planell-Morell et al., 2020). Although these records-based approaches carry statistical power and familiarity for real-world use, they take substantial time to consolidate. In times of disease outbreak, these traditional approaches therefore lose the immediate actionability needed to reduce negative consequences. Recent efforts focus on the use of wearable sensors to generate potential illness alerts more rapidly (Gadaleta et al., 2021; Richards et al., 2021; Mirjalali et al., 2022; Mason et al., 2022a). Although new screening approaches using wearable sensors hold great potential, they have yet to be validated for real-world use. Early results demonstrate that many algorithms created from wearable-collected data are burdened by a lack of physiological diversity, algorithmic generalizability, and unevenly distributed technology access (Futoma et al., 2020).

Web-based daily symptom survey tools could integrate with data collected in the field using wearable sensors as well as records-based data collected in more traditional ways. Web-based daily symptom surveys delivered via smartphones could rapidly reach large populations, thereby enabling mining to identify patterns indicating potential illness. By the same token, such surveys might allow classification of symptoms that correspond to likelihoods for different types of disease outbreaks (e.g., flu, COVID-19). These survey tools could also incorporate additional available demographic information (e.g., biological sex) to increase accuracy where different demographics have different manifestation of sickness. For example, despite substantial evidence for sex differences in COVID-19 outcomes (Gomez et al., 2021), we are unaware of efforts to incorporate these differences into COVID-19 screening efforts. Web-based daily symptom survey tools could therefore capitalize on rapid collection capabilities accessible to large populations and allow researchers to quickly incorporate emerging evidence (e.g., disease manifestation differences by sex) into optimized screening tools.

The first TemPredict study collected >4M daily symptom survey responses from a global participant pool of 63,153 individuals. We used these data to develop a Bayesian network to classify temporal windows for specific individuals as positive or negative for COVID-19, flu, or the cold, based on reported symptoms. Additionally, because participants provided demographic information in addition to their daily symptoms, we examined the impact of biological sex on the accuracy of COVID-19 detection. We report on the impact of biological sex on this classification network and highlight sex differences in symptom timing and likelihood. Taken together, these analyses hold implications for the development of web-based public health screening efforts.

## Methods

### Participants

**Figure 1:**
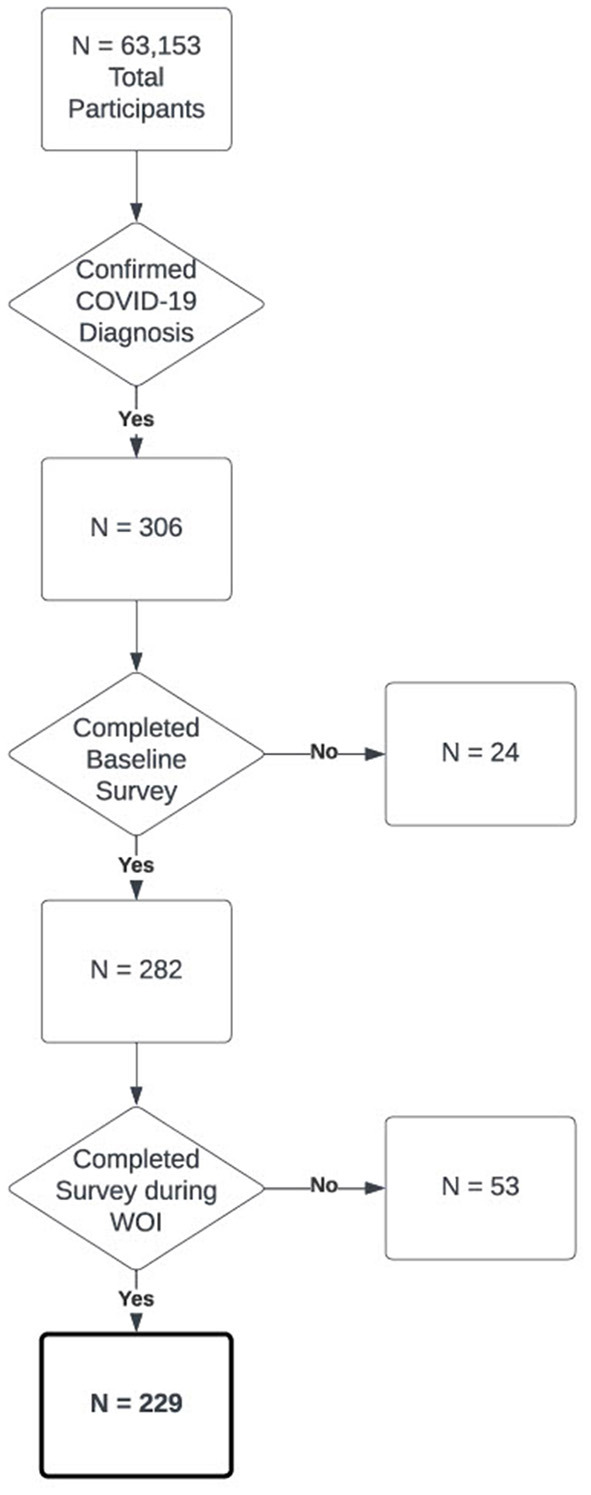
Flowchart of cohort selection for the COVID-19 positive cohort in this paper out of all participants in the model.

To identify positive COVID-19 cases amongst the 63,153 participants, we selected participants (*N* = 306) who met inclusion criteria for a confirmed COVID-19 diagnosis, and diagnosis date (DX date) which represented that a participant had completed a COVID-19 test that resulted in a positive result (Mason et al., 2022a). Of these 306 participants, 282 had completed a baseline survey requesting demographic information. Of these 282 participants, 53 did not provide web-based symptom survey responses from 3 weeks prior to 1 week after their DX date, which was the area of interest for our analysis. This resulted in a final cohort of 229 participants who had an identified COVID-19 diagnosis with an associated DX date as well as sufficient web-based symptom survey data (Figure 1).

Our negative control group was a group of participants who received a confirmed negative antibody test result (see Measures and Variable Preparation). We randomly selected 229 participants from the resultant 7,756 for whom we received negative antibody results (Ab-). The random sample was constrained to be age- and sex-matched to the COVID-19 positive cohort (see Supplementary Table S1 for exact composition of both cohorts). Symptom data for this cohort were limited to those reports captured within the preceding 3 months from the date of the Ab- test.

### Procedures

For a detailed description of study procedures, see (Mason et al., 2022a). In brief, participants enrolled in the TemPredict study (Smarr et al., 2020; Purawat et al., 2021; Mason et al., 2022a,b), sharing wearable device data and labels to help us develop COVID-19 detection capacities. Participants completed a single web-based baseline survey and, for as long as 8 months thereafter, had the opportunity to complete a web-based daily symptom survey that queried participants about acute illness symptoms typically observed in COVID-19 as well as any COVID-19 diagnoses. A subset of participants completed mail-based dried blood spot testing (see Mason et al., 2022a).

The University of California San Francisco (UCSF) Institutional Review Board (IRB, IRB# 20-30408) and the U.S. Department of Defense (DOD) Human Research Protections Office (HRPO, HRPO# E01877.1a) approved of all study activities, and all research was performed in accordance with relevant guidelines and regulations and the Declaration of Helsinki. All participants provided informed electronic consent. We did not compensate participants for participation.

### Measures and variable preparation

#### Demographic information

Participants provided demographic information including self-reported biological sex (“sex”) which we use as a comparison, and age which we use to balance across cohorts through the aforementioned web administered baseline survey that participants completed during study enrollment.

#### COVID-19 symptoms and diagnosis

Participants completed web-based daily symptom surveys that asked participants to report on a list of symptoms that characterize COVID-19 and other respiratory illnesses. The list of symptoms collected included the following: fever, chills, fatigue, general aches and pains, dry cough, sore throat, cough with mucus, cough with blood, shortness of breath, runny/stuffy nose, swollen/red eyes, headache, unexpected loss of smell or taste, loss of appetite, nausea/vomiting, and/or diarrhea (or “none of the above”). Participants responded using a multi-select format, and could not add other symptoms.

Participants also reported on COVID-19 test results, test dates, and test methods. Participants who reported a positive COVID-19 test result on an oral or nasopharyngeal swab, saliva, stool, or antigen test were included in the initial COVID-19 positive cohort. From these data we created the DX date variable, which was the earliest reported positive test date across surveys. If a participant reported multiple positive COVID-19 tests, we included only their first positive test (and first DX date). From these reports, we computed the following variables:

- *Prevalence:* Probability of a symptom being reported during a given instance of a specific illness.
- *Symptom rate:* Mean number of different symptoms that each participant endorsed per week.
- *Mean symptom rate:* Mean symptom rate across a group of participants.
- *Most common symptom:* Individual symptom most frequently reported per type of illness.
- *Window of interest (WOI):* Time interval defined by reference to the DX date in which a model is being assessed for accurate detection of illness. WOIs considered here range from: 3 weeks prior to DX date through 1 week post DX date.
- *Potential infection window (PIW):* Interval in time during which it was judged possible that an individual would have the onset of COVID-19 illness given the confirmation of virus detected by a PCR test on their DX date, as reported by them. This was judged to range from 3 weeks prior to DX date through to 3 weeks post DX date.

#### Antibody test kits and results

The antibody testing process was described in more detail previously (see Mason et al., 2022a). Briefly, we mailed kits for obtaining dried blood spots (TropBio Filter Paper Disks, Cellabs) to 10,021 participants and instructed participants to dry their blood spots overnight before returning by mail in plastic specimen bags containing a desiccant. We processed dry blood spots with eluent and tested using the Ortho Clinical Diagnostics VITROS^®^ SARS CoV-2 Total Assay.

### Analytic methods

#### Bayesian network construction

We trained Bayesian networks using the python package Pomegranate (Schreiber, 2018). A Bayesian network utilizes known properties of a system (for example, prevalence of illness symptoms) to generate a probabilistic relationship between a given set of symptoms and each disease class (for example, COVID-19, flu, and cold). The network can then be used to calculate probabilities of a participant having each disease based on a symptom profile (Chen and Pollino, 2012). We included each symptom as a node weighted by its prevalence in each specific illness (e.g., COVID-19, flu, or cold). We built an evidence matrix (matrix of node weights by symptom) from estimated symptom prevalence for each illness, including for COVID-19 in 2019 and 2020, from previous literature (Giacomelli et al., 2020; Wu et al., 2020; Yang et al., 2020). When specific symptoms in the matrix were exact matches to terms in the literature (e.g., dry cough vs. hacking cough), we chose one term for consistency. The symptom of “chills” was the only symptom that was not common in the COVID-19 literature, and we therefore set the prevalence to be that discovered in our data.

The input to the network is a list of Boolean values, indicating whether a participant endorsed a symptom during a given 7-day window. We tested a series of aggregate window sizes, and found that the 7-day window size optimized the true positive rate (TPR). The output from the network is presented as the probability distribution across all illnesses, with the maximum probability assigned as the illness that the network predicts.

#### Precision-recall

To generate precision recall metrics from the Bayesian network we split participants' data into three regions, the *positive region* which is the same as the POI, the *pre-negative region* which is all datapoints before the POI, and the *post-negative region* which is all datapoints past the POI. A positive prediction in each region is defined as at least one of the 7-day windows in the region being predicted as having COVID-19. In the context of precision-recall metrics, we define a true positive classification (not to be confused with the TPR as described in *Bayesian Network Construction*) as a positive prediction in the *positive region*, a false negative as a lack of positive prediction in the *positive region*, a true negative as a lack of a positive prediction in either of the negative regions, and a false positive as a positive prediction in either of the negative regions. Each of the negative regions were treated separately, so that, e.g., a person could have a false positive in their *pre-negative region* and a true negative in their *post-negative region*. From these counts we generated precision recall scores, where: precision = true positive/(true positive + false positive); recall = true positive/(true positive + false negative); F1 = 2 × (recall × precision)/(recall + precision).

#### Logistic regression methods

We trained basic logistic regression models using scikit-learn's (Pedregosa et al., 2018) basic Logistic Regression implementation. Logistic regression models iteratively fit a set of weights (beta coefficients) which multiply the input (in this case a participant's daily symptom profile) in order to maximize the number of correct predictions in its training data (Shipe et al., 2019). We extracted the beta coefficients from each model. A highly positive beta coefficient for a symptom indicated that the presence of that symptom contributes strongly to a COVID-19 prediction.

### Specific analyses

#### COVID-19 vs. flu vs. cold

We compared the mean symptom rate between COVID-19, flu, and cold, identifying a trend in the number of symptoms the participants endorsed for each disease during the PIW. To explore symptom progression, we visualized the proportion of people who reported a given symptom out of all the people that filled out a survey on that given day relative to DX date (i.e., day DX–5, as opposed to a date). We calculated this proportion for all symptoms during the WOI.

Using our Bayesian network, we compared COVID-19, flu, and cold illness manifestations by testing the network on all participants and assessing TPR (for COVID-19 detection) and false positive rate (FPR, for flu and cold). We report the percent of participants from each illness group that the model predicted as having COVID-19. To differentiate prediction from detection capacity, we calculated COVID-19 prediction probabilities for intervals starting with the 3 weeks leading up to the DX date, then incrementally adding one additional subsequent week (postdiagnosis) for 3 weeks (i.e., PIW).

To determine an FPR for COVID-19, we used antibody test results from the initial TemPredict study to identify a population of true negative participants. We used their prediction of COVID-19 as a metric of the FPR.

#### Differences in symptom presentations across biological sexes

We divided the COVID-19 population by sex, and then repeated the mean symptom rate analysis for each of the sexes separately. We trained several other Bayesian networks in the framework of sex as a biological variable, using different evidence matrices. In those cases, we either relied upon literature-reported values, or we generated “in house” evidence matrices based on symptom prevalence of the illnesses from our own data, grouped across sexes. Additionally, we generated male and female “in house” evidence matrices from the symptom prevalence of male and female participants separately (i.e., grouped within sexes).

We trained a Bayesian network with each of the above evidence matrices and calculated the percent of the male and female COVID-19 subpopulations that each network predicts as having COVID-19 at some point in the WOI. We compared the TPR of each network to see how sex affects each network's performance. We resampled 10 participants from each sex at a time, calculated the peak of each symptom for those 10 people in the PIW (calculated as the highest proportion of participants endorsing a symptom to the number of participants who completed a survey on a given day), resolving ties by taking the median. We repeated this 5,000 times for each sex, normalized the data for each symptom and sex by taking the kernel density estimate, setting the area under the curve of each symptom equal to one. This gave us a relative probability distribution for the timing of each symptom peak with regard to DX date.

We combined the COVID-19, flu, cold, and true negative cohorts into 2 separate datasets based on sex. Using a similar resampling approach, we trained a logistic regression model (as described in “Analytic Methods”) on a random 80% split of each dataset at a time, and saved the beta coefficients for each symptom. We repeated this 5,000 times for each sex.

## Results

### COVID-19 vs. flu vs. cold

Comparison of symptom prevalence across COVID-19, flu, and cold showed that people with COVID-19 experienced more symptoms than those ill with flu or cold, with a few exceptions, such as headaches, runny/stuffy nose, and nausea/vomiting (Table 1).

**Table 1:** The prevalence of each illness type and of reported symptoms per illness.

**Diagnosis**	**Other/No ID**	**COVID-19**	**Flu**	**Cold**
Prevalence	0.62	0.11	0.06	0.10
Chill	0.01	0.3	0.09	0.04
Fever	0.01	0.50	0.10	0.05
Fatigue	0.01	0.54	0.20	0.14
General aches/pain	0.01	0.30	0.13	0.09
Dry cough	0.01	0.63	0.16	0.10
Sore throat	0.01	0.20	0.20	0.12
Cough mucus	0.01	0.31	0.13	0.08
Cough blood	0.01	0.00	0.02	0.00
Short breath	0.01	0.37	0.09	0.06
Runny/stuffy nose	0.01	0.20	0.27	0.20
Swollen/red eyes	0.01	0.17	0.03	0.03
Headache	0.01	0.20	0.24	0.14
Loss of smell/taste	0.01	0.62	0.02	0.01
Loss of appetite	0.01	0.40	0.04	0.02
Nausea/vomiting	0.01	0.07	0.30	0.02
Diarrhea	0.01	0.14	0.09	0.03

COVID-19 rates were generated from pre-existing literature (see Measures and Variable Preparation), while cold and flu symptom prevalence were generated from in-house data.

**Figure 2:**
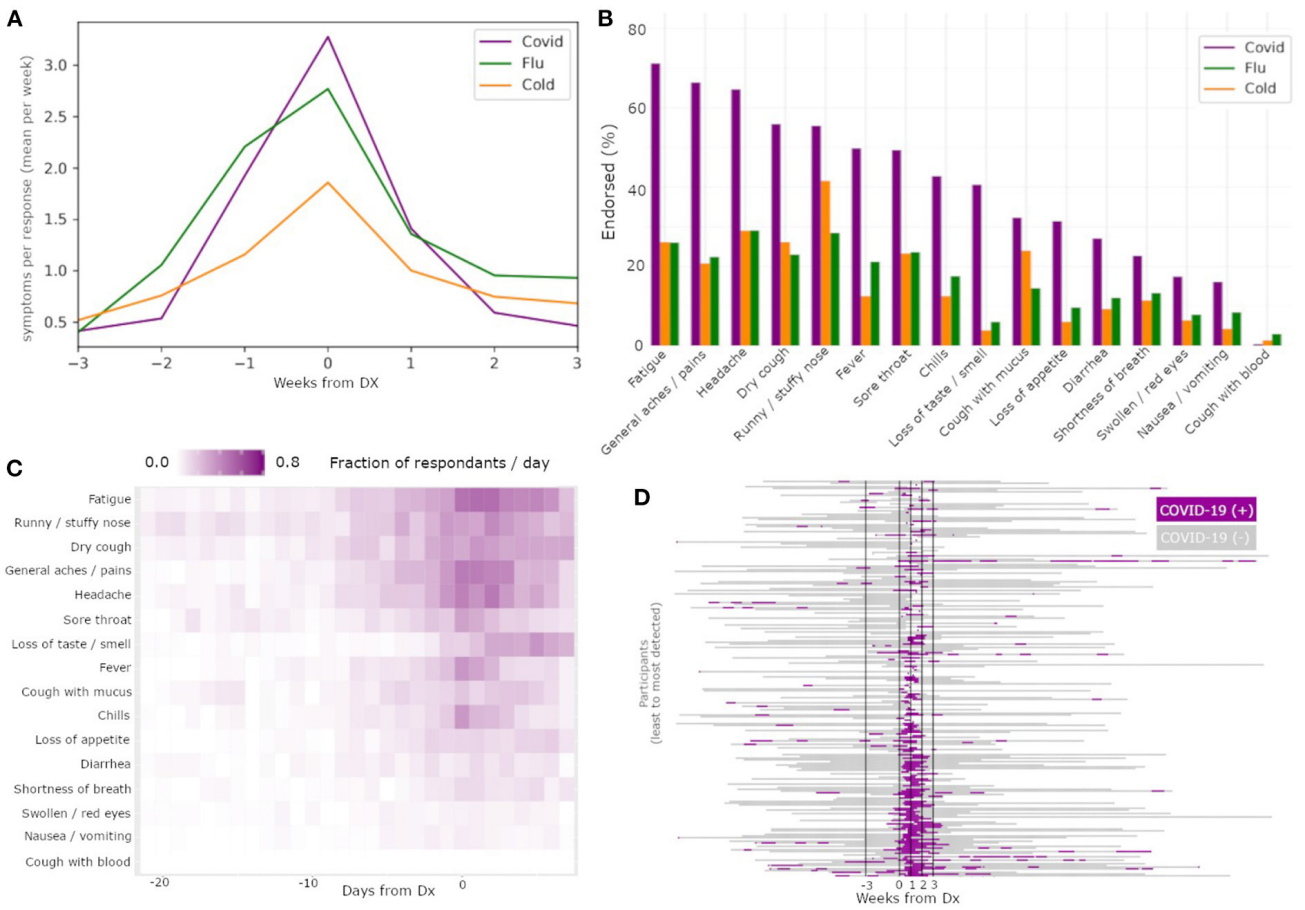
Differences among illnesses and across symptoms enable COVID-19 specific classification. The mean symptom rate for individuals with a diagnosis date reported for COVID-19 (yellow), flu (green), and cold (purple) **(A)**. Percent of individuals with these diagnoses reporting each of the recorded symptoms, by illness type, sorted by prevalence within COVID-19 subjects **(B)**. Heatmap of symptom report frequency by days from diagnosis **(C)** reveal different temporal profiles and likelihood across symptoms within reports paired to COVID-19 diagnoses. Results from a Bayesian classifier network **(D)** run on all times with symptom reports reveal good alignment between positive detections (green) and diagnosis date.

Symptom rates also differed between COVID-19 and those of flu and cold (Figure 2A). Specifically, COVID-19 had the largest number of symptoms endorsed per report, followed by flu, and then cold. Centering symptom reports around diagnosis date for all three illnesses resulted in clear peaks of reports, confirming that people experienced the most symptoms around the time of diagnosis. We also found differences in symptom prevalence between the three illnesses (Figure 2B). Fatigue was the most common symptom in COVID-19, while runny/stuffy nose was the most common symptom in both flu and cold. Notably, COVID-19 and flu each had several symptoms with similarly high rates of endorsement per reported illness.

The frequency of specific symptom endorsements relative to the DX date for COVID-19 (Figure 2C) revealed substantial differences in the duration and order of symptoms reported. Of note among these symptom profiles, fatigue had the highest area under the curve among the symptoms. Additionally, while loss of smell or taste has been reported as one of the most COVID-specific symptoms among the three illnesses (Dawson et al., 2021), in our data we observed participants reported loss of smell or taste later on average than other symptoms, limiting its usefulness in early illness screening despite its high COVID-19 specificity.

The Bayesian network had a TPR for COVID-19 detection of 71.6% in the WOI, and 78.16% in PIW (Figure 2D). Conversely, for flu, the network had a FPR for COVID-19 detection of 16.5% in the WOI, and 34.1% in the PIW. For cold, the network had an FPR for COVID-19 detection of 17.8% in the WOI, and 36.4% in the PIW (Table 2). The network had a precision score of 73.2%, recall of 71.6%, and F1 measure of 72.4%.

**Table 2:** The percent of individuals predicted as having COVID-19 by the Bayesian network run over an increasing number of weeks.

**Weeks**	**COVID-19—TP**	**Cold—FP**	**Flu—FP**	**Negative (FPR)**
−3: 0	34.5	9.27	5.8	5.6
−3: 1	71.6	17.8	16.5	6.1
−3: 2	77.7	28.5	27.1	6.5
−3: 3	78.16	36.4	34.1	8.7

Intervals began covering from 3 weeks prior to DX (−3) to DX (0). Intervals were expanded by increments of 1 week until they covered through to 3 weeks subsequent to DX (+3). For the COVID-19 positive group, this is the number of correct predictions (TP, %); for the cold, flu, and true negative cohort (FPR) this is the number of false predictions (FP, %).

### Sex differences

**Figure 3:**
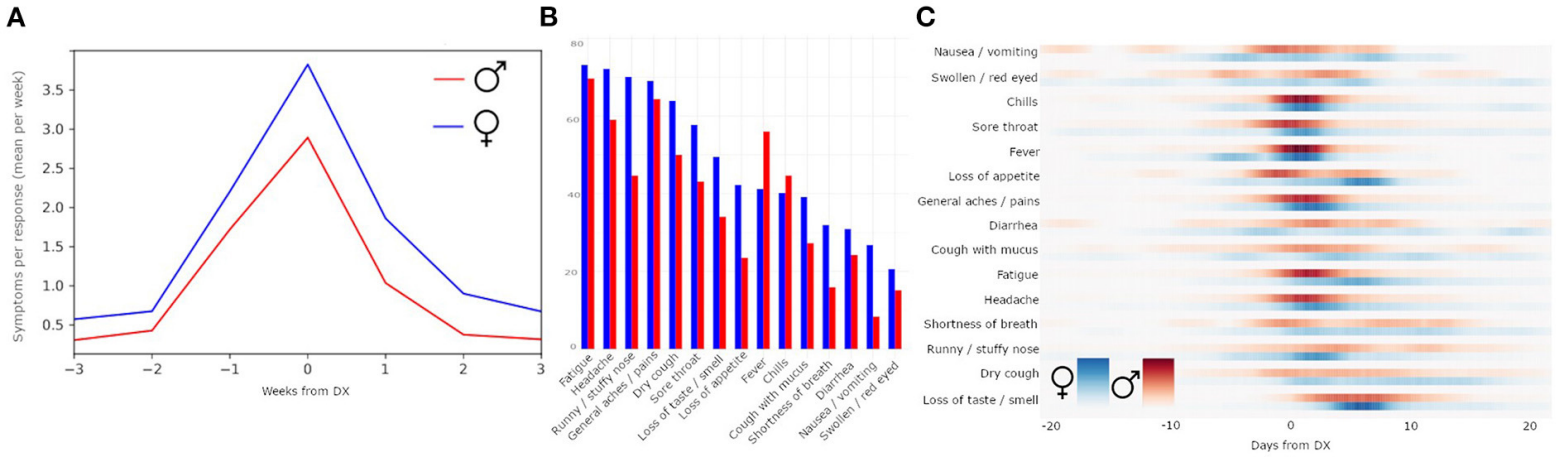
Sex differences in symptom reporting. Within individuals reporting COVID-19 diagnosis, females have a higher average mean symptom rate than males **(A)**. Comparison by individual symptoms reported **(B)** shows that symptom prevalence is not identical across sexes. The probability distribution of the peaks of symptoms relative to DX date, separated by females (blue) and males (red) **(C)**.

Comparison of the COVID-19 symptom rates indicated that the timing of the number of symptoms experienced by females and males was similar. However, females with COVID-19 had a higher mean symptom rate (Figure 3A; mean peak rate males = 2.89; mean peak rate females = 3.83); symptoms' prevalence were also higher in females than males, except “fever” and “chills” (Figure 3B; Table 3).

**Table 3:** Prevalence (% reporting) of symptoms in the COVID-19+ cohort for a given sex.

**Sex**	**Female**	**Male**
Fatigue	73.20	69.70
Headache	72.16	59.09
Runny/stuffy nose	70.10	44.70
General aches and pains	69.07	64.39
Dry cough	63.92	50.00
Sore throat	57.73	43.18
Loss of smell or taste	49.48	34.09
Loss of appetite	42.27	23.48
Fever	41.24	56.06
Chill	40.21	44.70
Cough mucus	39.18	27.27
Shortness of breath	31.96	15.90
Diarrhea	30.93	24.24
Nausea vomiting	26.80	8.33
Swollen/red eyes	20.62	15.15
Cough blood	1.03	0.00

Based on these observed differences, we re-evaluated the Bayesian network for each sex (Table 4). The network performed best for both sexes when trained on the external literature-reported (Giacomelli et al., 2020; Wu et al., 2020; Yang et al., 2020) data (male TPR: 66.7%; female TPR: 76.3%). The network trained on female data performed better on males (TPR = 64.4%) than the network trained on male data (TPR = 62.9%), while the network trained on male data performed better on females (TPR = 75.3%) than the network trained on female data (TPR = 74.4%). The networks were, on average, 10.25% more sensitive for COVID-19 in females than in males (Table 4).

**Table 4:** Bayesian models were trained on one of the following sources of symptom prevalence, described in Methods: literature search; in house female data; in house male data; combined sexes in house data.

**TPRs (%)**	**Model: 1** **literature**	**Model 2:** **female**	**Model 3:** **male**	**Model 4:** **combined** **sexes**
Male subgroup	66.7	64.4	62.9	65.2
Female subgroup	76.3	74.4	75.3	74.2

TPRs were then calculated for each model across male and female data separately.

The probability distribution of the peaks of each symptom, separated by sex, revealed that symptoms such as swollen/red eyes and nausea/vomiting had diffused peaks, while symptoms such as chills and general aches/pains had concentrated peaks (Figure 3C). Peaks in fatigue, general aches and pains, and loss of appetite came earlier for males than for females, whereas peaks for runny/stuffy nose came earlier for females than males. The symptom of dry cough was relatively equally distributed across males and females across time.

**Figure 4:**
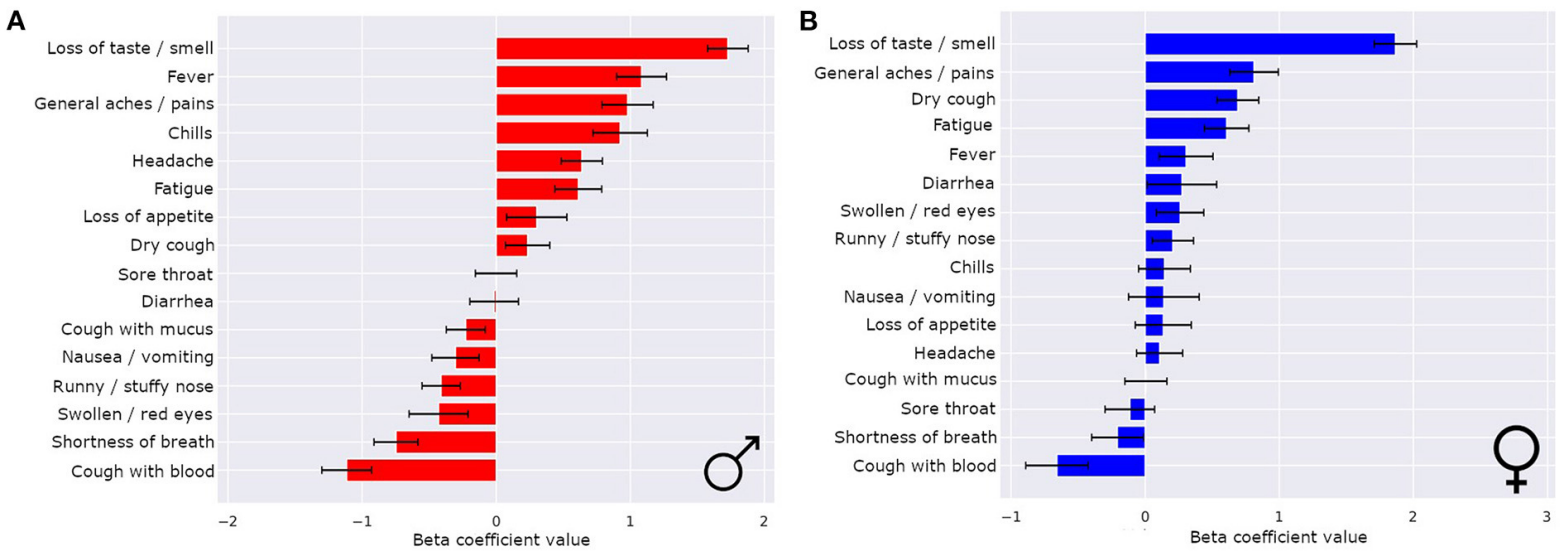
Sex differences in COVID-19 classifiers enables improved performance and symptom importance in both sexes. The means ± 1 std dev for the beta coefficients from logistic regression bootstrapping for males **(A)** and females **(B)**.

The symptoms that most strongly correlated with a COVID-19 illness were different for males and females (Figures 4A,B). Even though loss of smell or taste was the strongest indicator of COVID-19 for both sexes by a wide margin, there were more nuanced differences in the hierarchy of the rest of the symptoms. For males, the top four most indicative symptoms of COVID-19 after loss of smell or taste were fever, general aches/pains, chills, and headaches (in that order). For females, the top four most indicative symptoms of COVID-19 after loss of smell or taste were general aches/pains, dry cough, fatigue, and fever (in that order). Notably, for females, runny/stuffy nose was a positive indicator of COVID-19, whereas for males it indicated flu or cold over COVID-19.

## Discussion

### Principal results

Here we demonstrated that self-report symptom data gathered from large, distributed populations using web-based daily symptom surveys can be used to create predictive models for onset of COVID-19, with some ability to differentiate from other respiratory illnesses, including influenza (flu) and the common cold. We feel that such models can be trained to have sufficient precision that it is time to consider their utility in real-world screening efforts. Results reported here highlight the promise of predictive models that use differences between subpopulations of respondents to increase their accuracy, as we show here by highlighting differences by sex. In comparing sexes, we found that our symptom-based models could generate ranked lists of those symptoms most likely to be informative for someone being screened from a specific subpopulation (i.e., a patient being female or male affects interpretation of their symptoms).

There remains a sex imbalance in the development of screening tools, with women often disproportionately underrepresented (Dijkstra et al., 2008; Day et al., 2016; Larrazabal et al., 2020). Our work shows that there is no additional burden to gather data on both sexes, and that treating sex as a biological variable reveals meaningful differences that may improve screening efforts for both sexes. The finding that a model we derived of these data performed better in detecting COVID-19 on females than males across evidence matrices could reflect increased symptom reporting rate in females as compared to males. Future work should further assess the sex difference we observed in our symptom reports.

Our symptom profile data appear consistent with previously observed real-world data (Giacomelli et al., 2020; Wu et al., 2020; Yang et al., 2020), and support the hypothesis that symptoms alone could be sufficient to separate different illnesses in large scale screening efforts. In fact, our model reached its best results for an evidence matrix generated from the published literature (references of where we got the symptom list), as opposed to the evidence matrix derived from our own data set. This indicates that we did not overfit our model to the data, and further reinforces the idea that the network is capturing differences in symptoms appropriate to COVID-19. This supports the notion that there is a shared profile of symptoms that characterize COVID-19 and that we can detect these symptoms using web-based daily symptom surveys; moreover, these symptoms differ from those reported by individuals who are ill with flu or cold. Our findings support the hypothesis that web-based daily symptom survey methodology could be a relatively inexpensive and rapid tool to augment public health situational awareness.

In this context, we would like to remind those interested that classical metrics (specifically precision, recall, and F1 measure) can be misleading in the context of a Bayesian network. The capacity of such a network would be to serve as a screening tool, not a classification (i.e., diagnostic) tool. For example, from the point of view of a screening tool, one positive hit within a window of potential illness for an individual might be enough to be helpful, whereas classical precision and recall would define only one positive hit within the window of potential illness as failing to classify every illness day successfully. The result could be precision and recall scores that do not correlate well-with real-world usefulness.

### Limitations

We collected data between March, 2020 and November, 2020. As a result, the symptoms reported most likely all correspond to the alpha variant of COVID-19, which was the dominant variant at that time. Although results about the specific symptoms observed are therefore most relevant to that original variant, these analyses lay key groundwork for approaching the development of algorithms to predict subsequent COVID-19 variants (e.g., Delta, Omicron) or other respiratory illnesses.

One potential concern for using symptom screening in COVID-19 is the potential to miss asymptomatic individuals. Our model does not address this concern, and so might be more useful deployed over populations than as a reliable screening tool aimed specifically at individuals. Furthermore, while we show evidence that different illnesses can be classified successfully, the FPRs are still higher than is desired for reliable real-world discrimination among illnesses. Further research should evaluate how to rapidly implement surveys to support real-world public health efforts for specific populations and disease outbreaks. Given the low cost of deploying such methods over large populations, we expect that with proper development, the added information for public health surveillance and individual decision support (e.g., seeking a test or isolation) would be valuable even with this caveat.

Finally, this analysis was done with a limited sample size of 229 COVID-19 positive participants. This limitation was to ensure that all 229 had confirmed COVID-19 cases, rather than other illnesses, and to ensure availability of sufficient self-report survey data. Repeating our approach using larger samples of COVID-19 positive individuals would allow for broader symptom modeling comparisons. Additionally, such larger populations could be used to evaluate symptom profile differences between other groupings beyond sex, as in ethnicity, age, urban vs. rural living, etcetera.

## Conclusion

Symptom reports from web-based daily symptom surveys collected via smartphones provide sufficient information to build COVID-19 screening models. These models have the potential to discriminate between illnesses based on symptom prevalence and timing. Such surveys can be deployed inexpensively and broadly, making them an excellent candidate for development to support public health efforts as well as, in some cases, possibly useful tools for individual screenings (as in indications for getting a confirmatory test). Combining such efforts with more diverse populations, and integrating other sources of information (e.g., electronic medical records) could contribute to more nimble, discriminative, and widely-deployable public health tools for use in response to future pandemic outbreaks.

## Data availability statement

The raw data supporting the conclusions of this article will be made available by the authors, without undue reservation.

## Ethics statement

The studies involving human participants were reviewed and approved by the University of California San Francisco (UCSF) Institutional Review Board (IRB, IRB# 20-30408) and the U.S. Department of Defense (DOD) Human Research Protections Office (HRPO, HRPO# E01877.1a). The patients/participants provided their written informed consent to participate in this study.

## Author contributions

AK led analysis. AK, KP, and BS carried out experimental design. AK and BS carried out analysis and figure presentation. KP, SD, WH, AC, CA, LP, FH, AM, and BS carried out data gathering and survey design. All authors contributed to writing and editing the manuscript.

## Funding

The USAMRDC under the Department of Defense (DOD) provided financial support for this work. This effort was funded under MTEC solicitation MTEC-20-12-Diagnostics-023 and is funded by the USAMRDC under the Department of Defense. This effort is also based upon work supported by the Department of the Army under Air Force Contract No. FA8702-15-D-0001 and the US Department of Defense Air Force Office of Scientific Research, through the Massachusetts Institute of Technology Lincon Laboratory. The #StartSmall foundation provided financial support for this work. Oura Health Oy provided 1,400 pieces of hardware and financial support in the form of a sponsored research contract.

## Conflict of interest

Authors BS and AM have received compensation within the last 12 months from OuraRing Inc. The remaining authors declare that the research was conducted in the absence of any commercial or financial relationships that could be construed as a potential conflict of interest.

## Publisher's note

All claims expressed in this article are solely those of the authors and do not necessarily represent those of their affiliated organizations, or those of the publisher, the editors and the reviewers. Any product that may be evaluated in this article, or claim that may be made by its manufacturer, is not guaranteed or endorsed by the publisher.

## Author disclaimer

The views and conclusions contained herein are those of the authors and should not be interpreted as necessarily representing the official policies or endorsements, either expressed or implied, of the U.S. Government.
