# Supplementary material for: Methods for detecting probable COVID-19 cases from large-scale survey data also reveal probable sex differences in symptom profiles
Source: Front Big Data. 2022 Nov 10;5:1043704. doi: 10.3389/fdata.2022.1043704 (PMC9685297; doi:10.3389/fdata.2022.1043704)
Supplement: Supplementary file 1 [file Table_1.DOCX]

**Supplemental materials.**

**Supplemental Table 1.**

Percentage of participants by 10-year age bracket and by sex, for the COVID-19 positive cohort (COVID-19+) and the negative control cohort selected from participants with negative antibody test kit results (Ab-).

| **Age / Sex** | **COVID-19+** | **Ab-** |
| --- | --- | --- |
| 11-20 | 0.4 | 0.9 |
| 21-30 | 15.3 | 15.3 |
| 31-40 | 24.5 | 26.6 |
| 41-50 | 26.2 | 27.5 |
| 51-60 | 22.7 | 21 |
| 61-70 | 9.2 | 7.9 |
| 71-80 | 1.7 | 0.9 |
| 81-90 | 0 | 0 |
| Male | 62 | 57.6 |
| Female | 38 | 42.4 |
